# Characteristics of inflammatory mediators in dental pulp inflammation and the potential for their control

**DOI:** 10.3389/fdmed.2024.1426887

**Published:** 2024-08-06

**Authors:** Nobuyuki Kawashima, Takashi Okiji

**Affiliations:** Department of Pulp Biology and Endodontics, Division of Oral Health Sciences, Graduate School of Medical and Dental Sciences, Tokyo Medical and Dental University (TMDU), Tokyo, Japan

**Keywords:** inflammation, mediators, dental pulp, inflammatory cells, inflammatory control

## Abstract

Dental pulp is a mesenchymal connective tissue located inside the rigid encasement of the dentin. When bacteria or bacterial products invade the dental pulp, inflammation known as pulpitis is induced in this tissue. Various mediators produced during the course of pulpitis profoundly modify the pathophysiology of the inflammation. Typical mediators include cytokines, chemokines, nitric oxide, reactive oxygen species, matrix metalloproteinases, proteases, neutrophil extracellular traps, neuropeptides, and eicosanoids. Controlling these mediators may potentially lead to the healing of pulpitis and the preservation of pulp tissue. This review discusses these mediators and further explores the possibility of controlling them.

## 1 Introduction

Dental pulp is a nerve- and vascular-rich mesenchymal connective tissue surrounded by a mineralized tissue called dentin (1). Cells with fibroblastic characteristics (known as dental pulp cells) are the major cellular constituents of the pulp tissue. Along the most peripheral portion of the pulp tissue subjacent to the pulp−dentin border, a layer of cells responsible for dentin formation, known as odontoblasts, are located. The pulp tissue also contains dental pulp stem cells, which are mesenchymal stem cells that have the ability to differentiate into a new generation of odontoblast-like cells responsible for reparative dentin formation when the original odontoblasts are lost as a result of severe damage to the pulp (2). Various types of immunocompetent cells are also present in the dental pulp tissue. In particular, dendritic cells constitutively expressing major histocompatibility complex class II molecules are abundant just below the odontoblast layer and respond as antigen-presenting cells to bacterial stimuli derived via the dentin tubules (3, 4). Class II-negative resident macrophages, another major immune cell population of the pulp, are diffusely distributed throughout the pulp tissue. These cells express M2 macrophage markers such as CD163 and may be associated with the repair of injured pulp tissue by promoting angiogenesis (5). The nerves and blood vessels that are abundant in the pulp tissue also characterize the pathophysiology of this tissue.

Following invasion of bacterial stimuli into the pulp tissue, typically through a carious lesion or traumatically exposed dentin, localized pulp inflammation is initiated in the area around the site of the initial challenge in the coronal pulp. Pulp inflammation then spreads to the root pulp tissue, exhibiting variable histology and clinical manifestations. Clinically, pulpitis is classified as reversible pulpitis or irreversible pulpitis, the latter being further classified into symptomatic and asymptomatic irreversible pulpitis (6). Reversible pulpitis usually progresses to irreversible pulpitis, but the acute manifestation of irreversible pulpitis depends on the balance between the degree of bacterial invasion and the activity of the defense systems present in the pulp tissues (7).

Although the clinical classification of pulpitis cannot be strictly applied to the histopathologic classification, the nature of the inflammatory reaction occurring in pulpal inflammation is essentially no different from inflammation in other parts of the body. However, because the pulp is situated in a low-compliance environment surrounded by mineralized tissues, intrapulpal pressure increases with the progression of exudation causing compression of blood vessels to be shunted (8), impeding circulation in the pulp. In other words, the impaired circulation and accompanying hypoxia cause deep modifications of pulpal inflammation (9, 10).

To cope with exogenous noxious stimuli, the pulp tissue has the ability to self-protect against dentinal tubule-derived bacterial invasion by forming reparative dentin, and resident and recruited immune cells eliminate bacterial products that have invaded the pulp (1). Dense sensory innervation of the pulp indicates that, in addition to evoking pain as a warning signal, neurogenic inflammation may be deeply involved in the pathophysiological responses of this tissue; the neurogenic inflammation involves vasodilation and vascular permeability increases through the release of neuropeptides, such as substance P, from the endings of sensory nerve fibers (11). However, if bacterial infection from the oral cavity persists, pulpitis spreads from the coronal region to the apex. The pulp eventually becomes necrotic and the defense mechanisms of the pulp tissue are no longer triggered, ultimately leading to tooth loss.

Various inflammatory mediators produced during the course of pulpitis profoundly modify the pathophysiology of the inflammation. Many of these mediators are also produced in healthy pulp tissue, where they are associated with physiological functions and contribute to the maintenance of homeostasis of this tissue. This article aims to give an overview of the properties and roles of the key mediators of pulpitis and to explore the possibility of therapeutic control of these mediators.

## 2 Mediators in pulpitis

**Figure 1:**
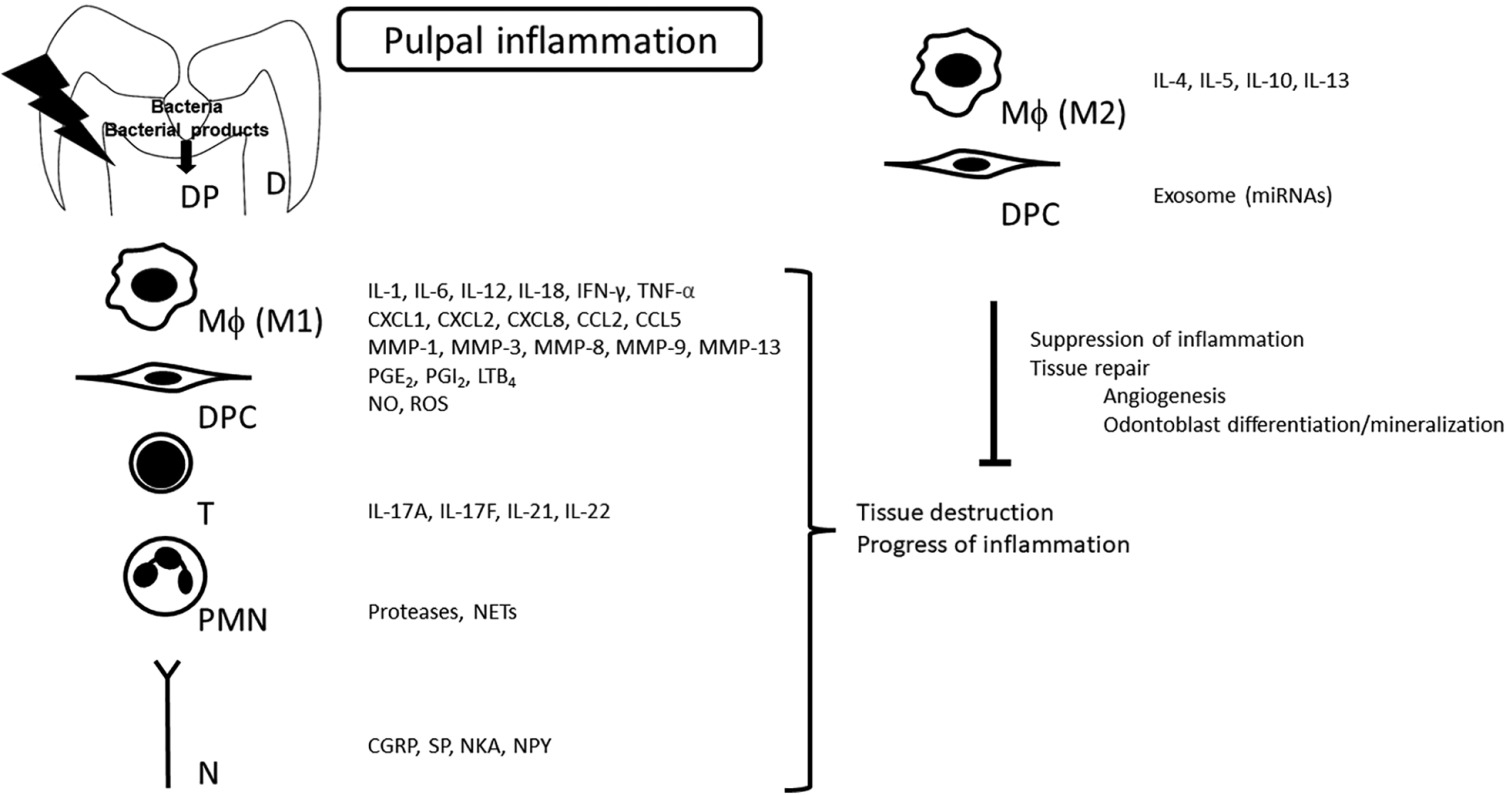
Synthesis of mediators in pulpal inflammation. Various mediators produced during the pulpal inflammatory process profoundly modulate the pathogenesis of pulpitis. At the same time, as inflammation progresses, the production of anti-inflammatory mediators is induced as a result of negative feedback, which is another characteristic of pulpitis. D, dentin; DP, dental pulp; Mϕ, macrophage; DPC, dental pulp cells; T, T cells; PMN, polymorphonuclear leukocyte; N, nerve; IL, interleukin; IFN, interferon; TNF, tumor necrosis factor; MMP, matrix metalloproteinase; PG, prostaglandin; LT, leukotriene; NETs, neutrophil extracellular traps; CGRP, calcitonin gene-related peptide; SP, substance P; NKA, neurokinin A; NPY, neuropeptide Y.

Mediators discussed in this review are listed in Figure 1.

## 3 Cytokines and chemokines

### 3.1 Proinflammatory cytokines

Immunocompetent cells such as macrophages eliminate bacteria and bacterial products that invade the pulp through the dentinal tubules, and activated macrophages produce various kinds of proinflammatory mediators (7). Typical proinflammatory cytokines such as interleukin (IL)-1, IL-6, IL-12, IL-18, interferon (IFN)-γ, and tumor necrosis factor (TNF)-α are predominantly synthesized by M1 macrophages in pulpal inflammation. Among these cytokines, elevated levels of IL-6 have been detected in various types of samples, including pulp tissue (12), pulp blood (13, 14), and dentinal fluid (15) obtained from inflamed pulps. This indicates that IL-6 is a candidate biomarker that can discriminate the condition of the pulp between healthy and irreversibly inflamed (16).

Clinically, pulpitis is often regarded as a chronic inflammatory lesion that may acutely transform when the body’s defense capabilities are compromised (17). Because of the chronic nature of pulpitis, a large number of T lymphocytes are found to accumulate in the inflamed pulp (18), and T lymphocyte-derived cytokines are also involved in the pathogenesis of pulpitis (7, 19). The principal Th1 effector cytokine is IFN-γ, which activates macrophages (20). Th17-derived cytokines include IL-17A, IL-17F, IL-21, and IL-22 (21), and the presence of IL-17 has been reported in inflamed pulp tissue (22).

### 3.2 Anti-inflammatory cytokines

Key Th2 cytokines include IL-4, IL-5, IL-10, and IL-13 (23). In particular, IL-10 possesses potent anti-inflammatory properties, and it plays a central role in limiting the host immune response to pathogens (24). IL-10 is also produced by macrophages of the M2 type, which are primarily involved in wound healing and tissue repair and are classified into several subtypes (M2a-d) (25). The dynamics of these cells in pulpitis have not yet been fully elucidated. Levels of IL-10 in pulp blood are significantly higher in samples from cariously exposed pulps and irreversibly inflamed pulps compared with those from normal pulps (Elsalhy et al., 2013). These higher levels may indicate that IL-10 acts to dampen any excessive inflammatory reaction before the establishment of irreversibly inflamed pulp status.

### 3.3 Chemokines

Cytokines with the property of inducing migration of inflammatory/immunocompetent cells are specifically referred to as chemokines. Chemokines are classified into four subclasses (CCL, CXCL, CX3CL, and XCL), and bind to G protein-coupled heptahelical chemokine receptors and induce migration of their target cells (26). Chemokines also possess key properties of macrophage activation and polarization to different functional phenotypes (M1, M2a, M2b, M2c) (27). Increased expression of IL-8, which is classified as a CXCL chemokine and is a powerful neutrophil chemoattractant, is detected in irreversibly inflamed human dental pulps (13, 28–31) indicating that IL-8 may be used as a biomarker for the diagnosis of irreversible pulpitis (16). In experimentally induced rat pulpitis, the kinetics of CXCL1 (GROa), CXCL2 (GROb), and CCL2 (MCP1) mRNA expression correlates with the infiltration of neutrophils, and CCL5 (RANTES) mRNA expression correlates with the infiltration of macrophages (32).

## 4 Nitric oxide, reactive oxygen species, and matrix metalloproteinases

Nitric oxide (NO), a small free radical, and reactive oxygen species (ROS) including superoxide and hydrogen peroxide, are effectors of the immune response and possess critical signaling roles in physiology and pathophysiology (33). The expression of nicotinamide adenine dinucleotide phosphate-diaphorase (NADPH-d), an indicator of nitric oxide synthase, is significantly higher in inflamed pulp tissues than in normal healthy pulp tissues (34). Acute pulpal inflammation in human teeth enhances the mRNA and protein levels of inducible nitric oxide synthase (iNOS) (35, 36), which acts as a key enzyme in inflammation and immune activation processes through the production of NO from L-arginine (37). ROS are molecules or ions formed by the incomplete one-electron reduction of oxygen and are key signaling molecules in the progress of inflammation (38). In particular, superoxide is involved in the regulation of autophagy (39).

Macrophage-derived proteases include subclasses of matrix metalloproteinases (MMPs), disintegrins and metalloproteinases (ADAMs), and TNF-α converting enzymes (TACE, ADAM17). MMPs are zinc-dependent proteases and are secreted by various types of cells, including fibroblasts, osteoblasts, endothelial cells, vascular smooth muscle cells, macrophages, neutrophils, lymphocytes, and cytotrophoblasts (40). MMPs are key enzymes in matrix degradation, and a functional balance between MMPs and tissue inhibitors of metalloproteinases regulates tissue degradation (41). An increase of MMP-1, MMP-8, and MMP-13 levels in chronic inflamed pulp has been observed (42). MMP-9, a neutrophil-derived MMP, is proposed as a local biomarker useful for distinguishing reversible and irreversible pulpitis because higher levels of MMP-9/total protein in pulpal fluid were significantly associated with the failure of direct pulp capping (43). However, the application of MMP-3 had anti-inflammatory effects on experimentally induced pulpitis in canines (44) and rats (45).

## 5 Proteases, neutrophil extracellular traps

Various proteases are produced during inflammation. When acute inflammation occurs, neutrophils infiltrate the front line of infection and are responsible for removing exogenous stimuli. Neutrophil-derived proteases include neutrophil-derived serine proteases (NSPs), neutrophil elastase (NE), and proteinase-3 (PR-3). Neutrophil proteases are mainly responsible for the intracellular killing of pathogens, but their extracellular release upon neutrophil activation is involved in tissue damage at the sites of inflammation (46). Neutrophils are equipped with bactericidal devices in the form of neutrophil extracellular traps (NETs). NETs consist of a meshwork of chromatin fibers composed of granule-derived antimicrobial peptides and enzymes such as neutrophil elastase, cathepsin G, and myeloperoxidase (47). NETs have been detected in inflamed pulp, and may contribute to disease progression (48).

## 6 Neuropeptides

Sensory nerves are abundantly distributed in the dental pulp tissue. Neuropeptides released from peripheral nerve terminals include calcitonin gene-related peptide (CGRP), substance P (SP), neurokinin A (NKA), vasoactive intestinal peptide (VIP), and neuropeptide Y (NPY). Increased expression of CGRP, SP, NKA, and NPY is detected in inflamed human pulp compared with that in healthy pulp (49). Furthermore, the mRNA expression of SP and its receptor, neurokinin-1 receptor, is detected in pulp fibroblasts (50). The local release of these vasoactive peptides is thought to cause neurogenic inflammation (51).

## 7 Eicosanoids

The eicosanoid family comprises 20-carbon polyunsaturated fatty acid metabolites (52). The classical eicosanoids, including prostaglandins (PGs), thromboxanes, leukotrienes (LTs), and hydroxy-, hydroperoxy-, epoxy- and oxo-eicosanoids, play a critical role in the regulation of inflammation (52). Elevated synthesis of PGE_2_, 6-keto-PGF_1_*_α_* (a stable metabolite of PGI_2_) (53), and LTB_4_ (54) is detected in experimentally induced rat dental pulp inflammation. An increase in the PGE_2_ level was detected in human pulps diagnosed as having reversible pulpitis (55). Eicosanoids are thought to essentially exacerbate inflammation, and the cyclooxygenase pathway involved in eicosanoid production is a major target for nonsteroidal anti-inflammatory drugs (NSAIDs) (56). Recently, the potential of these eicosanoids to induce the healing of pulpitis, including hard tissue induction, has been a focus for research. PGE_2_ alone is cytotoxic, but its inclusion in microspheres induces hard tissue marker expression in pulp cells (57). PGE_2_ induces cAMP production, which is mainly mediated by the EP2 receptor (58). Furthermore, EP2/EP4 agonists promote the generation of endothelial cell filopodia and upregulation of genes related to odontoblast differentiation (59). PGI_2_ induces MMP-9 production, which is expected to be involved in pulp tissue healing (60). Incorporation of LTB4 into the microsphere induces odontoblast differentiation and mineralization (61). Additionally, resolvin E1, an *ω*-3 polyunsaturated fatty acid (PUFA) metabolite and not an arachidonic acid metabolite, suppresses inflammatory mediator production and induces hard tissue marker expression in human dental pulp stem cells (DPSCs) (62).

## Discussion

In pulpal inflammation, macrophages are mainly responsible for the production of inflammatory and anti-inflammatory cytokines. Macrophages are classified as M1 and M2 (63), and M2 macrophages are attracting attention for their function in healing and the termination of inflammation (64). Conditioned medium from M2 macrophages induces odontoblast marker expression in DPSCs (65), and hard tissue marker expression in dental pulp cells mainly through TGF-β (66). M2 polarization is reported to be induced by conditioned medium from human DPSCs (67), GSK-3β inhibitor small molecules (68), and an injectable dental pulp-derived decellularized matrix hydrogel (69). Calcium silicate materials, including mineral trioxide aggregate (MTA), which is currently the most popular direct pulp capping material, are reported to cause M2 polarization *in vitro* (70) and *in vivo* (71). Further studies on the development of methods and materials to induce M2 macrophage polarization and migration in pulp tissue are expected to be conducted.

MicroRNAs (miRNAs), which comprise a highly conserved group of small, non-coding RNA molecules, regulate gene expression primarily by silencing, and they play essential roles in the physiology and development of cells and tissues (72). We revealed that microRNA (miR)-21 and miR-146b are highly expressed in experimentally induced rat pulpitis and lipopolysaccharide-stimulated human dental pulp cells, and both miRNAs possess anti-inflammatory effects via downregulation of the NF-kB signaling pathway, a major cascade of proinflammatory mediator synthesis (73, 74). Various miRNAs involved in pulpal inflammation, including miR-21, have been reviewed by Muñoz-Carrillo et al. (75). Application of these miRNA mimics induces downregulation of proinflammatory signals/medications, which are the targets of pulpal inflammation control. Currently, the use of exosomal miRNAs is widely debated in relation to their therapeutic potential (76). Exosomes are cell-derived extracellular vesicles that promote cell−cell communication, and they may be used as suitable carriers for miRNA delivery.

Detecting mediators may allow us to infer the state of inflammation (77). The amount of MMP-9 in the leachate from the pulp surface is reported to be a promising biomarker that can indicate the inflammatory state of the pulp tissue (43). However, the relationship between the MMP-9 levels and pathological changes in the pulp is still unclear. Further research should be directed to identify appropriate biomarkers to evaluate the inflammatory state of the pulp and clearly show that the amount of the biomarker(s) correlates with the pathology of pulpitis.

Suppression of proinflammatory mediator production accompanied by elimination of bacterial infection seems to induce healing of pulpal inflammation. In experimentally induced rat pulpitis, application of a specific iNOS inhibitor can reduce macrophage infiltration into the pulp tissue and decrease the mRNA expression of pro-inflammatory cytokines and cyclooxygenase-2 (78, 79). However, inflammatory mediators may play a positive role as well as a negative one; some induce healing and promote the differentiation of odontoblasts (80). The transition to healing may be induced by the suppression of excessive production rather than complete suppression. Optimal regulation of mediator synthesis opens the way for healing of pulpal inflammation and recovery of the integrity of pulpal tissue (Figure 1).
